# Neck and upper extremity pain in sonographers – a longitudinal study

**DOI:** 10.1186/s12891-020-3096-9

**Published:** 2020-03-12

**Authors:** Jenny Gremark Simonsen, Anna Axmon, Catarina Nordander, Inger Arvidsson

**Affiliations:** 1 Division of Occupational and Environmental Medicine, Lund University, SE-223 81, Lund, Sweden; 2 Division of Occupational and Environmental Medicine, EPI@LUND (Epidemiology, Population studies, and Infrastructures at Lund University), Lund University, SE-223 81, Lund, Sweden

**Keywords:** Diagnostic imaging, Physical, Psychosocial, Visual ergonomics, Women, Working conditions, Ultrasonography

## Abstract

**Background:**

Sonographers have reported a high occurrence of musculoskeletal pain for more than 25 years. Assessments of occupational risk factors have previously been based on cross-sectional surveys. The aim of this longitudinal study was to determine which factors at baseline that were associated with neck/shoulder and elbow/hand pain at follow-up.

**Methods:**

A questionnaire was answered by 248 female sonographers at baseline and follow-up (85% of the original cohort). 208 were included in the analyses. Physical, visual, and psychosocial work-related conditions were assessed at baseline. Pain in two body regions (neck/shoulders and elbows/hands) was assessed at both baseline and follow up.

**Results:**

Pain at baseline showed the strongest association with pain at follow-up in both body regions [prevalence ratio (PR) 2.04; 95% confidence interval (CI) 1.50–2.76], for neck/shoulders and (PR 3.45; CI 2.29–5.22) for elbows/hands. Neck/shoulder pain at follow-up was associated with inability of ergonomic adjustments at the ultrasound device (PR 1.25; CI 1.05–1.49), a high mechanical exposure index (PR 1.66; CI 1.09–2.52), and adverse visual conditions (PR 1.24; CI 1.00–1.54) at baseline. Moreover, among participants with no neck/shoulder pain at baseline, high job demands (PR 1.78; CI 1.01–3.12), and a high mechanical exposure index (PR 2.0; CI 0.98–4.14) predicted pain at follow-up. Pain in the elbows/hands at follow-up was associated with high sensory demands at baseline (PR 1.63; CI 1.08–2.45), and among participants without pain at baseline high sensory demands predicted elbow/hand pain at follow-up (PR 3.34; CI 1.53–7.31).

**Conclusion:**

Pain at baseline was the strongest predictor for pain at follow-up in both body regions. We also found several occupational factors at baseline that were associated with pain at follow-up: inability to adjust equipment, adverse visual conditions, a high MEI, high job demands and high sensory demands. These results point at a possibility to influence pain with better ergonomics.

## Background

Sonographers are skilled health care professionals who perform ultrasound examinations [1]. Such examinations are becoming increasingly common due to improved technology and knowledge, and a growing need [2]. However, conducting sonography is associated with risk factors for work-related musculoskeletal disorders (WMSDs) such as awkward postures and sustained static forces [3–5]. Furthermore, sonography is a computer-intensive work task since both the examination and the ensuing analysis are performed on a computer, constituting yet another risk factor for WMSDs [6]. Further, high occurrence of WMSDs has been reported among sonographers over more than 25 years [7].

In a previous cross-sectional study, we investigated the associations between reported pain and a number of occupational factors among 291 sonographers [8]. Pain was assessed in two regions; the neck/shoulders and the elbows/hands. For both body regions we found a positive association between pain and computer-related eye complaints, high job demands, high sensory demands and a high self-reported mechanical exposure (MEI; postures and movements). Additionally, in a larger cohort, including nurses, teachers and the sonographers, the sonographers reported a higher prevalence of shoulder pain than the other groups, using the same outcome measure of pain [9]. Considering these results, it is urgent to identify and determine which occupational factors that are not only associated but predictive of pain. Consequently, a follow-up questionnaire was sent to the 291 sonographers about two and a half years after the initial study.

The aim of this study was thus to determine which factors at baseline that were associated with neck/shoulder and elbow/hand pain at follow-up, in sonographers.

## Methods

### Study design

This was a longitudinal questionnaire study, collecting data on exposure at baseline and outcome at follow-up. A questionnaire on working conditions, ergonomic and visual conditions, physical- and psychosocial workload, personal characteristics and musculoskeletal pain was distributed to Swedish female sonographers at baseline (March 2010 through October 2012) and at follow-up (September 2012 through April 2015 [mean follow-up time 29 months; SD 2]).

### Study population

At baseline, a cohort of 291 female sonographers employed in all the clinical physiology and cardiology departments in hospitals throughout Sweden (*n* = 45), answered a self-administered questionnaire [8]. The inclusion criteria were: working at least 20 h per week and performing sonography for at least four hours per week during the previous three months before filling out the questionnaire. A follow-up questionnaire was sent to the cohort about two and a half years after baseline. Of the 291 participants at baseline, 248 (85%) answered to the follow-up questionnaire i.e. 43 participants did not respond at follow-up. Among those who responded, 40 (16%) were excluded because they no longer fulfilled the inclusion criteria concerning ongoing sonographic work (retired, parental leave or changed work, < 4 h sonography/week). Thus, 208 (71% of the original cohort) sonographers were included in the follow-up study (Table 1).

**Table 1:** Neck/shoulder and elbow/hand pain in sonographers at baseline and follow-up

	Neck/shoulders			Elbows/hands		
		Pain at baseline	Pain at follow-up		Pain at baseline	Pain at follow-up
	*N*	*N* (%)	*N* (%)	*N*	*N* (%)	*N* (%)
*Participants at baseline*	289	169 (58)		291	85 (30)	
*Participants at follow-up*	205	125 (61)	140 (68)	208	63 (30)	65 (31)
*Excluded at follow-up*	43			40		
Missing outcome data at follow-up	4	2 (50)		0	–	
Retired or on parental leave	12	7 (58)	4 (33)	12	3 (25)	1 (8)
No longer fulfilled inclusion criteria^a^	27	17 (63)	13 (48)	28	12 (43)	6 (21)
*Non-responders*	41	16 (39)		42	6 (14)	

^a^ Other kinds of leave > 50%, changed work or < 4 h/ ultrasound week

### Data collection

At baseline and at follow-up the participants answered a questionnaire. The follow-up questionnaire which was a modified version of the questionnaire used at baseline [8] included questions on personal characteristics [10], working conditions, ergonomic and visual conditions [8], physical- and psychosocial workload [11–15] and musculoskeletal pain [16–19]. Detailed information about all exposure variables are given in Table 2.

**Table 2:** Exposure variables (questionnaire) including questions on exposure, answers/options and how the analysis was performed

Item	Question on exposure	Answers/options	For analysis
Personal Characteristics	Age		Trichotomized: 23–37; 38–53; 54–66 years
	Height		Trichotomized: 153–163; 164–174; 175–185 cm
	Weight		Trichotomized: 45–65, 66–86, 87–107 kg
			BMI dichotomized: 17.8–24.9; 25–29.9 kg/m^2^
Personal Characteristics	Hours/day of personal recovery time [10]	Hardly any time at all; < 1 h/day; 1 h/day; 2 h/day; 3 h/day; ≥4 h/day.	Dichotomized: ≤2; ≥3 h/day
Personal Characteristics	Frequency of physical exercise [10]	Never; Occasionally; Once a week, twice to four times/week; five times/week or more	Dichotomized ≤ once; ≥ twice a week
Personal Characteristics	Hours of household work [10]	0–2; 3–10; 11–20; 21–30; ≥ 31 h/week	Dichotomized: ≤ 10; ≥ 11 h/week
Working conditions	Years as a professional sonographer [8]		Dichotomized at the median ≤ 13 years; > 13 years
Working conditions	Hours of work/week? [8] Hours of sonography/week? [8]		Hours/week was dichotomized as part-time (< 37 h/week) or full-time work (≥ 37 h/week) Sonography/week was dichotomized at the median ≤ 18 h/week; > 18 h/week As these were highly correlated competition was performed including both variables in the same model, and only the factor giving the lowest *p*-value was included in the main analyses (working hours/week for the neck/shoulders and sonography hours/week for the elbows/hands)
Echocardiography	Do you perform echocardiography?	Yes/no	Dichotomized
Ergonomic conditions during examinations with the ultrasound device	Possibility to adjust a) screen height b) screen tilt), c) the keyboard, d) the chair e) table for examinations [8]	Yes/no to all	When positive answer to all items we considered that ergonomic adjustments were possible
Ergonomic conditions during computer work	Satisfaction with the computer work station arrangements? [8]	Very satisfied (can work comfortably); Rather satisfied; Neither satisfied or dissatisfied; Rather dissatisfied; Very dissatisfied (uncomfortable/strenuous work)	Trichotomized: very/rather satisfied; neutral; rather/very dissatisfied
Ergonomic conditions	Performing examinations in the ward on in-patients (bedside examinations)? [8]	Never; Seldom; Sometimes; Daily	Dichotomized: Never/seldom; sometimes/daily
Visual conditions	a. Eyestrain related to computer work? [8] b. Headache related to computer work? [8] c. Eyesight adequately corrected? [8]	a and b) Never; Seldom; Sometimes; Often; Very often. c) Good/adequately corrected; Inadequately corrected	Good visual conditions were considered present if the person had no headaches or eye complaints related to sonography examinations or computer work and sufficiently corrected eyesight
Physical workload	11 items regarding awkward work postures, Mechanical exposure Index (MEI) [11, 12]	Each item answered on a three-point scale Almost not at all; Some; A lot	The total score was calculated for each individual (range 11–33). MEI (sum score) was dichotomized at unexposed/low (≤15) and medium/high exposure (> 15)
Psychosocial conditions	The Job Content Questionnaire was used regarding job demands (nine items), job control (nine items), job support (eight items) [13, 14]	Each item answered on a four-point scale indicating the degree of agreement. Higher values on the scale indicate higher demands, better control and better support.	The mean value in each dimension was calculated for each individual. Each dimension was dichotomized at low/high based on the median value.
Psychosocial conditions	One dimension of The Copenhagen Psychosocial Questionnaire: sensory demands (five items) [15]	Each item was answered on a five-point scale. Higher values indicated higher demands.	The mean value in each dimension was calculated for each individual. The dimension was dichotomized at low/high based on the median value.

To assess the outcome, i.e. musculoskeletal pain at follow up, the participants were asked about subjective musculoskeletal complaints (aches, pain or discomfort) in the neck, shoulders, elbows and hands during the past 12 months using the Nordic Questionnaire [15]. In addition for each body region, information was collected about the frequency of complaints during the past year, using a 5-point scale (never, seldom, sometimes, often or very often) [16], as well as the intensity of complaints on an eleven-point scale from 0 (none at all) to 10 (extremely severe) [17]. We considered pain to be present if the participant reported complaints at least “seldom” with an intensity of at least 7 (very severe), “sometimes” with an intensity of at least 3 (moderate), or “often” or “very often” with an intensity of at least 2 (slight/mild) [18]. The body regions were merged together into the two separate regions neck/shoulder and elbow/hand. Pain was defined for each region.

### Statistical methods

We used multivariate models with exposure at baseline as independent variables and pain at follow-up as dependent variables. Predictors of development of pain or recovery from pain were assessed in two different ways. 1) By including pain at baseline as an independent variable, and 2) by analysing data stratified by pain at baseline. Prevalence ratios (PRs) with 95% confidence intervals (CIs) were estimated by Poisson regression. The statistical package for the social sciences, SPSS 22 (IBM SPSS Statistics, 22 Commuter License, Armonk, New York, USA), was used.

#### Selection of confounders

Personal characteristics (age, height, weight, body mass index BMI, personal recovery time, physical exercise and house hold work) were assessed as potential confounders in a two-step procedure. As there were only four smokers we did not consider smoking as a characteristic.

The selection of confounders was done separately for each body region. In the first step, pair-wise associations were assessed with the outcome variable (pain). Potential confounders with *p* < 0.20 in these analyses were carried forward to the second step, in which they were assessed against the different exposure variables one by one. Potential confounders that were associated with at least one exposure (p < 0.20) were included in the model building.

#### Model building

Separate models to identify associations between exposure at baseline and pain at follow up were created for each outcome. Firstly we assessed the pair-wise association between each exposure and each outcome (Model 1). Secondly, these associations were assessed adjusting for the confounders selected according to the procedure described above (Model 2). All exposures (excluding pain at baseline) that were associated with the outcome (*p* < 0.05) were included in a multivariate model, which also comprised the confounders (Model 3). Finally, the multivariate analyses were adjusted for (Model 4) pain at baseline and confounders. In Model 5 we assessed pair-wise associations for conditions statistically significant in Model 3, stratified by pain at baseline and adjusted for confounders. Thus, the five models were

- Model 1: Pair-wise associations, unadjusted
- Model 2: Pair-wise associations, adjusted for confounders
- Model 3: Multivariate model with exposures with *p* < 0.05 in model 2, adjusted for confounders
- Model 4: Model 3, adjusted for pain at baseline and confounders
- Model 5: Model 3, pair-wise associations stratified by pain at baseline and adjusted for confounders

## Results

Two hundred and eight (71% of the original cohort) sonographers were included in the follow-up analyses. Their mean age at baseline was 45 years (range 24–65; SD: 12) (Table 1).

### Changes in the presence of pain between baseline and follow-up

**Fig. 1:**
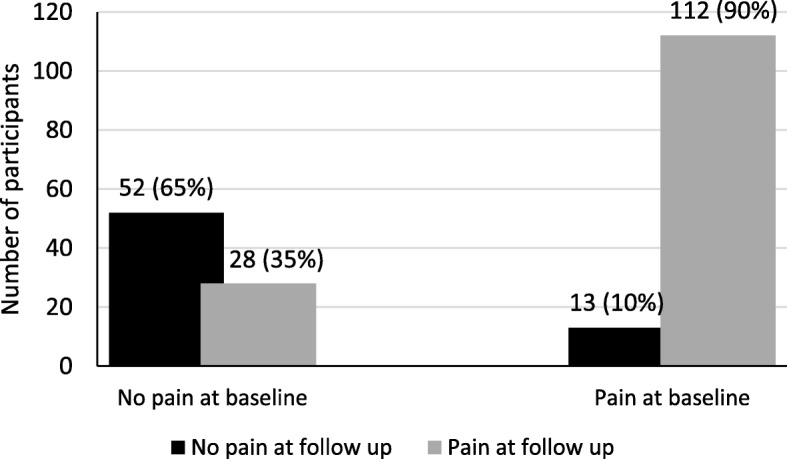
Health status at follow-up among participants without (*N* = 80) and with (*N* = 125) pain in the neck/shoulders at baseline

The overall prevalence of neck/shoulder pain increased from 61% (*N* = 125), to 68% (*N* = 140), during the follow-up period (Fig. 1). Among the sonographers who reported pain at baseline (N = 125), 112 (90%) also did so at follow-up. Twenty-eight (35%) of the 80 who did not report pain at baseline reported pain at follow-up.

**Fig. 2:**
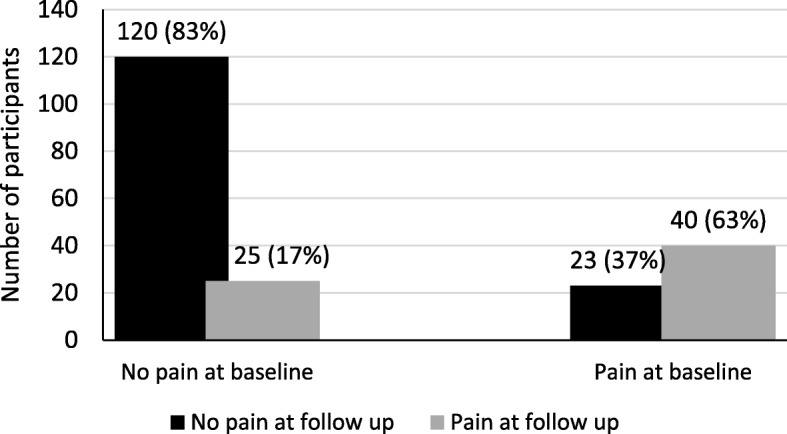
Health status at follow-up among participants without (*N* = 145) and with (*N* = 63) pain in the elbows/hands at baseline

Among the sonographers who reported elbow/hand pain the prevalence of pain increased from 30% (*N* = 63) to 31% (*N* = 65) during the follow-up period (Fig. 2). Among the participants who reported pain at baseline (N = 63), 40 (63%) also did so at follow-up (Fig. 2). Twenty-five (17%) of the 145 that did not report pain at baseline reported it at follow-up.

### Associations between exposures at baseline and pain at follow-up

In the pair-wise models (Model 1 and 2; Table 3), pain at baseline, inability of ergonomic adjustments at the ultrasound device, adverse visual conditions, dissatisfaction with the computer workstation arrangements, a high MEI and high job demands were associated with neck/shoulder pain at follow-up. In the multivariate model (Model 3) associations remained for inability of ergonomic adjustments, poor visual conditions and a high MEI.

**Table 3:** Associations between self-reported factors at baseline and musculoskeletal pain in the neck/shoulders at follow-up

		Pain at follow-up	Model 1^a^		Model 2^b^		Model 3^c^		Model 4^d^	
	*N*	*N* (%)	PR	CI	PR	CI	PR	CI	PR	CI
Neck/shoulder pain at baseline										
No pain	80	28 (35)	1		1				**1**	
Pain	125	112 (90)	**2.56**	**(1.88–3.47)**	**2.54**	**(1.88–3.42)**			**2.04**	**(1.50–2.76)**
Years as a professional sonographer										
0.1–13	110	76 (69)	1		1					
13.1–36	94	63 (67)	0.97	(0.80–1.17)	0.96	(0.79–1.16)				
Working hours/week										
20–36	96	59 (61)	1		1					
37–41	109	81 (74)	1.21	(0.99–1.47)	1.19	(0.97–1.46)				
Echocardiography										
No	54	39 (72)	1		1					
Yes	151	101 (66)	1.08	(0.89–1.31)	1.10	(0.89–1.35)				
Bedside examinations										
No/Seldom	142	92 (65)	1		1					
A few times per week/Daily	62	47 (76)	1.17	(0.97–1.41)	1.18	(0.98–1.42)				
Ergonomic adjustments possible										
Yes	116	71 (61)	1		1		1		1	
No	85	68 (80)	**1.31**	**(1.10–1.56)**	**1.33**	**(1.11–1.59)**	**1.25**	**(1.05–1.49)**	1.13	(0.97–1.32)
Good visual conditions										
Yes	79	44 (55)	1		1		1			
No	122	94 (77)	**1.38**	**(1.11–1.72)**	**1.43**	**(1.15–1.78)**	**1.24**	**(1.00–1.54)**		
Satisfaction with computer workstation arrangements										
Very satisfied/Rather satisfied	125	80 (64)	1		1		1		1	
Neutral	47	32 68)	1.04	(0.89–1.22)	1.04	(0.89–1.21)	1.04	(0.84–1.29)	1.03	(0.87–1.23)
Rather/Very dissatisfied	28	25 (90)	**1.29**	**(1.11–1.48)**	**1.25**	**(1.08–1.44)**	1.06	(0.87–1.29)	1.01	(0.85–1.21)
MEI (mechanical exposure index)										
Unexposed/Low (11–15)	30	12 (40)	1		1		1		1	
Medium/High (16–33)	165	124 (75)	**1.88**	**(1.20–2.94)**	**1.94**	**(1.27–2.99)**	**1.66**	**(1.09–2.52)**	1.34	(0.90–1.99)
Job demands (cut-off: 2.44)										
Low	105	61 (58)	1		1					
High	99	79 (80)	**1.37**	**(1.14–1.66)**	**1.38**	**(1.15–1.67)**	1.13	(0.94–1.36)		
Job control (cut-off: 2.83)										
Low	112	83 (74)	1		1					
High	92	57 (62)	0.84	(0.69–1.02)	0.84	(0.70–1.10)				
Job support (cut-off: 2.87)										
Low	120	89 (74)	1		1					
High	84	39 (46)	0.82	(0.67–1.00)	0.85	(0.68–1.02)				
Sensory demands (cut-off: 80)										
Low	55	34 (62)	1		1				1	
High	149	106 (71)	1.09	(0.91–1.32)	1.15	(0.91–1.32)			1.12	(0.95–1.33)

Calculated with Poisson regression as the prevalence ratio (PR) and 95% confidence interval (CI). Results in boldface are statistically significant

^a^ Pair-wise, unadjusted

^b^ Pair- wise, adjusted for BMI and physical exercise

^c^ Multivariate model with exposures with p < 0.05 in model 2, adjusted for BMI and physical exercise

^d^ Multivariate model with exposures with *p* < 0.05 in model 2, adjusted for BMI and physical exercise and for pain at baseline

For elbows/hands, pain at baseline and high sensory demands were associated with pain at follow-up (Model 1 and 2; Table 4). The association with high sensory demands remained in the multivariate model (Model 3).

**Table 4:** Associations between self-reported factors at baseline and musculoskeletal pain in elbows/hands at follow-up

		Pain at follow-up	Model 1^a^ (unadjusted)		Model 2^b^		Model 3^c^		Model 4^d^	
	N	N (%)	PR	CI	PR	CI	PR	CI	PR	CI
Elbow/hand pain at baseline										
No pain	145	25 (17)	1		1				1	
Pain	63	40 (63)	**3.68**	**(2.46–5.51)**	**3.60**	**(2.40–5.42)**			**3.45**	**(2.29–5.22)**
Years as a professional sonographer										
0.1–13	110	32 (29)	1		1					
13.1–36	98	32 (33)	1.13	(0.76–1.70)	1.43	(0.86–2.39)				
Sonography (h/week)										
1–18	105	31(30)	1		1					
19–40	103	34 (33)	1.12	(0.75–1.67)	1.19	(0.80–1.77)				
Echocardiography										
No	56	17 (31)	1		1					
Yes	152	48 (32)	0.96	(0.61–1.52)	0.96	(0.61–1.51)				
Bedside examinations										
No/Seldom	145	42 (29)	1		1					
A few times per week/Daily	62	22 (35)	1.23	(0.81–1.87)	1.20	(0.79–1.83)				
Ergonomic adjustments possible										
Yes	117	36 (31)	1		1					
No	87	29 (33)	1.08	(0.72–1.62)	1.09	(0.74–1.62)				
Good visual conditions										
Yes	79	26 (33)	1		1					
No	125	38 (30)	0.92	(0.61–1.39)	0.87	(0.58–1.31)				
Satisfaction with computer workstation arrangements										
Very satisfied/Rather satisfied	128	39 (30)	1		1					
Neutral	47	16 (34)	1.12	(0.69–1.80)	1.13	(0.71–1.80)				
Rather/Very dissatisfied	28	9 (32)	1.02	(0.58–1.92)	1.13	(0.63–2.05)				
MEI										
Unexposed/Low (11–15)	33	6 (18)	1		1					
Medium/High (16–33)	165	57 (63)	1.90	(0.89–4.04)	1.87	(0.89–3.95)				
Job demands (cut-off: 2.44)										
Low	108	29 (27)	1		1					
High	100	36 (36)	1.32	(0.86–1.99)	1.30	(0.87–1.94)				
Job control (cut-off: 2.83)										
Low	113	39 (35)	1		1					
High	95	26 (27)	0.80	(0.53–1.21)	0.83	(0.55–1.26)				
Job support (cut-off: 2.87)										
Low	122	43 (35)	1		1					
High	86	22 (26)	0.73	(0.48–1.13)	0.76	(0.49–1.16)				
Sensory demands (cut-off: 80)										
Low	112	27 (24)	1		1		1		**1**	
High	95	38 (40)	**1.65**	**(1.10–2.50)**	**1.63**	**(1.08–2.45)**	**1.63**	**(1.08–2.45)**	**1.46**	**(1.00–2.12)**

Calculated with Poisson regression as the prevalence ratio (PR) and 95% confidence interval (CI). Results in boldface are statistically significant

^a^ Pair-wise, unadjusted

^b^ Pair- wise, adjusted for height and physical exercise

^c^ Multivariate model with exposures with p < 0.05 in model 2, adjusted for height and physical exercise

^d^ Multivariate model with exposures with p < 0.05 in model 2, adjusted for height and physical exercise and for pain at baseline

When adjusting for pain at baseline (Model 4), only pain at baseline remained a significant predictor of neck/shoulder pain at follow-up. For elbows/hands, pain at baseline and high sensory demands predicted pain at follow-up.

### Predictors of pain or recovery

Predictors of pain or recovery were evaluated using Model 5, i.e. stratifying by pain at baseline. Among those who did not report pain at baseline, a high MEI (almost statistically significant) and high job demands predicted neck/shoulder pain (Table 5). High sensory demands was a significant predictor for elbow/hand pain. Among those who reported pain at baseline we found no significant predictors of persistent pain or recovery.

**Table 5:** Associations between self-reported factors at baseline and pain at follow-up, stratified by pain at baseline

	Model 5							
	Pain at baseline				No pain at baseline			
	N	%	PR	CI	N	%	PR	CI
*NECK/SHOULDERS*^*a*^								
Ergonomic adjustments possible								
Yes	63	(51)	1		53	(67)	1	
No	61	(49)	1.10	(0.98–1.24)	24	(30)	1.49	(0.85–2.58)
Visual conditions								
Yes	35	(29)	1		44	(56)	1	
No	87	(71)	1.13	(0.96–1.32)	35	(44)	1.14	(0.65–2.02)
Satisfaction with the computer work-station arrangements								
Very satisfied /Rather satisfied	71	(58)	1		54	(68)	1	
Neutral	28	(23)	1.08	(0.99–1.19)	19	(30)	0.86	(0.69–1.08)
Rather/very dissatisfied	23	(19)	0.98	(0.79–1.22)	5	(2)	1.20	(0.78–1.85)
Mechanical exposure index score								
Unexposed/low (11–15 p)	9	(7)	1				1	
Medium/High (16–33 p)	111	(93)	1.19	(0.84–1.69)			2.01	(0.98–4.14)
Job demands (cut-off: 2.44)								
Low	56	(55)	1		49	(62)	1	
High	69	(45)	1.11	(0.98–1.26)	30	(38)	**1.78**	**(1.01–3.12)**
*ELBOWS/HANDS*^*b*^								
Sensory demands (cut-off: 80)								
Low	30	(48)	1		82	(56)	**1**	
High	33	(52)	0.90	(0.62–1.30)	62	(43)	**3.34**	**(1.53–7.31)**

Pair-wise associations for conditions that were statistically significant in Model 3 (Tables 3 and 4), calculated with Poisson regression as the prevalence ratio (PR) and 95% confidence interval (CI). Results in bold face are statistically significant

^a^Adjusted for BMI and physical exercise

^b^Adjusted for height and physical exercise

## Discussion

### Principal findings

Unsurprisingly, pain at baseline was the factor that showed the strongest association with pain at follow-up in both body regions. Inability of ergonomic adjustments at the ultrasound device, adverse visual conditions, a high MEI and pain at baseline were also associated with neck/shoulder pain at follow-up, but when adjusting for pain at baseline these associations were no longer statistically significant. This may be explained by the fact that there was a strong association between ergonomic/visual conditions and pain already at baseline [8], and there was no further increase of the associations at follow-up. Among those who did not report pain at baseline, high job demands predicted incidence of pain at follow-up.

High sensory demands at baseline was associated with elbow/hand pain at follow-up. This association remained both after adjustment for pain at baseline and when analysing only among those who did not report pain at baseline.

### Strengths and limitations

The most important strength is the longitudinal design. To our knowledge, this is the first longitudinal study that aims at exploring associations between occupational factors and pain among sonographers. Another strength with the present study was that the outcome criteria included a combination of frequency and intensity of pain. Our definition focuses on complaints that are of a certain dignity, which give a higher relevance than to just ask whether the subject has experienced any pain during the past year.

A limitation was that we only performed two enquiries with a quite long interval. The study design is insensitive to fluctuations that may occur after baseline up to twelve months before follow up. Still, the results concerning which factors at baseline that are associated with pain at follow up are valid. Responders at follow-up reported a higher frequency of pain at baseline than the non-responders, possibly reflecting an unhealthy worker selection bias. However, as the response rate at follow up was as high as 85% we believe the effect of such selection bias, if present, to be minor.

Individuals with pain are more inclined to overestimate their exposure due to the pain, which may result in an incorrect association between exposure and pain [20, 21]. Concerning factors that are objective and not influenced by the person’s own experience, such as whether the equipment is possible to adjust, we do not expect such information bias. Job demands and high sensory demands, which are based on self-reports of own experience, predicted pain among participants that did not meet the criteria for pain at baseline. Thus, pain did not affect their perception of demands at baseline. Accordingly, on the whole, we do not believe that our results are affected to any major extent by information bias due to pain at baseline.

Traditionally, causal associations are investigated by following healthy previously unexposed participants over time. However, though the cohort included more than 200 participants, only 80 of them did not report neck/shoulder pain at baseline, which limited the possibility of studying causal associations using traditional methods. Further, they had already worked as sonographer for in median 13 years, prior to this study, (range 0.25–36 years), and some of those that were prone to develop pain due to adverse working conditions were possibly already affected. To be able to utilize all the information in the study population we applied several strategies. Associations between pain and working conditions at baseline could include pain that was present throughout the study. Adjustment for pain at baseline enabled us to detect the effect of change in pain between baseline and follow-up (either incidence or recovery). Thus, we consider model 3, 4 and 5 to all contribute with valuable information concerning which factors at work that are relevant to consider in preventive actions.

### Musculoskeletal pain

The pain definition used in the present study has so far seldom been reported, and it is therefore challenging to compare the prevalence of pain among the sonographers with that in the general population. However, in a study of highly exposed Swedish dental personnel the prevalence of neck/shoulder pain was 56% using the same case definition [22]. Therefore, we consider the baseline prevalence of 61% in the present study to be high. Further, in a large cohort study, including the sonographers in the present study but also teachers and nurses, pain in shoulders was more common among the sonographers than among the other groups and the prevalence of shoulder pain increased more from baseline to follow-up among the sonographers than it did in the other occupational groups (Arvidsson I, Nordander C, Gremark Simonsen J, Lindegård Andersson A, Björk J: The impact of occupational and personal factors on musculoskeletal pain - A longitudinal cohort study of female teachers, nurses and sonographers, unpublished). In a large cohort study concerning the working population in France, an incidence of episodic neck pain, i.e. neck pain at least eight days during the previous twelve months, was 15% over five years in previously pain free women [23]. In our study, 35% among the sonographers that did not report neck/shoulder pain at baseline did so at follow-up, already after two and a half years. This indicates that sonographers are more prone than workers in general to develop neck/shoulder pain.

The majority of the sonographers with neck/shoulder pain at baseline were still affected at follow-up, and the number of participants with pain increased. Pain is a well-known risk factor for future pain [24] and pain at baseline was the most notable predictor for pain at follow-up in both body regions. The participants in our cohort had however worked as sonographers for in median 13 years and the high prevalence of pain already at baseline may to some extent be due to associations with present or former occupational risk factors. The optimal study would be to follow workers from their first day in the occupation, but that has not been possible for us.

### Recommendations

The sonographers showed a high prevalence of pain in neck and upper limb and were exposed to several well-known ergonomic and organisational risk factors. Optimizing working condition may reduce pain and based on the associations at baseline as well as on knowledge from the literature [4, 5, 25–30] we would like to make some recommendations. We recommend the equipment to be possible to adjust to fit the examiner’s anthropometrics and to allow variation in work technique. Also, the sonographers should be encouraged to vary their postures and movements.

Previous studies have reported that adverse visual conditions cause musculoskeletal discomfort [31–33]. Sonographers often turn the light on and off during and between examinations, and it takes longer to adapt from one lighting level to another with increasing age [32]. Sonographers have also reported that perceived poor lighting, e.g. glare or dazzle in the screen, leads to eyestrain and musculoskeletal discomfort [5]. There is limited evidence for the effects of visual interventions to prevent visual symptoms among computer users [34]. However, the sonographers’ visual conditions include extraordinary conditions and we thus believe improvements to be important.

To reduce job demands, we recommend organisational efforts to prevent stress from e.g. unplanned or prolonged examinations, delays and technical errors [35].

In summary, the sonographers´ workplace needs a multidisciplinary approach, involving both ergonomists and optometrists [36], since the arranging is complex, taking into account anthropometric measures and age.

## Conclusion

Pain at baseline was the strongest predictor for pain at follow-up in both body regions. We also found several occupational factors at baseline that were associated with pain at follow-up: inability to adjust equipment, adverse visual conditions, a high MEI, high job demands and high sensory demands. These results point at a possibility to influence pain with better ergonomics. Future research should aim at exploring whether these factors represent a cause and effect relationship. Further, intervention studies should be done to test whether the recommended ergonomic or organizational changes impact future pain.

## Data Availability

A copy of the questionnaire can be obtained from the corresponding author. The datasets used and/or analysed during the current study are available from the corresponding author on reasonable request.

## Abbreviations

BMI: Body Mass Index
CI: Confidence Interval
MEI: Mechanical exposure index
PR: Prevalence Ratio
WMSD: Work related musculoskeletal disorders
